# Use of auto‐germ to model germination timing in the sagebrush‐steppe

**DOI:** 10.1002/ece3.4591

**Published:** 2018-11-14

**Authors:** William C. Richardson, Dallin R. Whitaker, Kyler P. Sant, Nicholas S. Barney, Ryan S. Call, Bruce A. Roundy, Zachary T. Aanderud, Matthew D. Madsen

**Affiliations:** ^1^ Department of Plant and Wildlife Sciences Brigham Young University Provo Utah

**Keywords:** germination rate, restoration, seeding, thermal time, wet‐thermal accumulation model

## Abstract

Germination timing has a strong influence on direct seeding efforts, and therefore is a closely tracked demographic stage in a wide variety of wildland and agricultural settings. Predictive seed germination models, based on soil moisture and temperature data in the seed zone are an efficient method of estimating germination timing. We utilized Visual Basic for Applications (VBA) to create Auto‐Germ, which is an Excel workbook that allows a user to estimate field germination timing based on wet‐thermal accumulation models and field temperature and soil moisture data. To demonstrate the capabilities of Auto‐Germ, we calculated various germination indices and modeled germination timing for 11 different species, across 6 years, and 10 *Artemisia*‐steppe sites in the Great Basin of North America to identify the planting date required for 50% or more of the simulated population to germinate in spring (1 March or later), which is when conditions are predicted to be more conducive for plant establishment. Both between and within the species, germination models indicated that there was high temporal and spatial variability in the planting date required for spring germination to occur. However, some general trends were identified, with species falling roughly into three categories, where seeds could be planted on average in either fall (*Artemisia tridentata* ssp. *wyomingensis* and *Leymus cinereus*), early winter (*Festuca idahoensis, Poa secunda*,* Elymus lanceolatus*,* Elymus elymoides*, and *Linum lewisii*), or mid‐winter (*Achillea millefolium*,* Elymus wawawaiensis*, and *Pseudoroegneria spicata*) and still not run the risk of germination during winter. These predictions made through Auto‐Germ demonstrate that fall may not be an optimal time period for sowing seeds for most non‐dormant species if the desired goal is to have seeds germinate in spring.

## 1 INTRODUCTION

Seed germination timing strongly impacts the success of direct seeding efforts in wildland systems by influencing exposure to pathogens, nutrients and soil moisture, temperature, light, herbivory, and other biotic and abiotic factors (Gornish et al., 2015; James & Carrick, 2016). For these reasons, several studies have tracked germination timing in the field to better understand and improve seeding outcomes (Abbott & Roundy, 2003; Boyd & James, 2013; Gerrit, 1991; James, Rinella, & Svejcar, 2012). However, tracking seed germination timing in the field can be challenging, resource intensive, and time‐consuming. Additionally, knowledge gained from short‐term field germination studies is often lacking due to high annual variability in weather conditions at the time of the experiment (Hardegree, Jones, Roundy, Shaw, & Monaco, 2016). Subsequently, to gain general inferences from germination studies, labor‐intensive studies need to be repeated for multiple years.

Researchers have turned to predictive germination models for a more efficient method of estimating germination timing (Allen, Benech‐Arnold, Batlla, & Bradford, 2007; Bradford, 2002; Hardegree, Moffet, Walters, Sheley, & Flerchinger, 2017; Hardegree & Van Vactor, 1999). In recent years, models have been developed that assume there are naturally occurring processes within the seeds themselves already in place to regulate germination timing (Finch‐Savage & Leubner‐Metzger, 2006). It has been shown that the majority of these processes are a function of temperature and moisture (Allen, Debaene‐Gill, & Meyer, 1992; Bradford, 1990; Hardegree, Jones, Pierson, Clark, & Flerchinger, 2008; Hardegree, Van Vactor, Pierson, & Palmquist, 1999).

Progress toward germination for many cool‐season species can be predicted through a wet‐thermal accumulation model where soil moisture must exceed a base water potential (*Ψ*_b_) for germination to occur (Finch‐Savage, Steckel, & Phelps, 1998; Rawlins, 2009; Rawlins, Roundy, Davis, & Egget, 2012; Roundy, Hardegree, Chambers, & Whittake, 2007). The base water potential used is derived through laboratory experimentation (Roundy et al., 2007). Though there are many factors that influence the rate of seed germination and number of germinable seeds, adjusting *Ψ*_b_ is expected to correct for impacts from environmental conditions, after‐ripening and seasonal changes in dormancy cycling (Bradford, 2002). Subsequently, once *Ψ*_b_ is determined, seed germination timing and number of germinable seeds may be accurately predicted from soil temperature. Field trials have validated wet‐thermal accumulation models (Rawlins, Roundy, Egget, & Cline, 2012; Rawlins, Roundy, Davis et al., 2012), and confirmed their utility in predicting seed germination in a number of settings, with a wide variety of species (Cline, Roundy, & Christensen, 2018a,b; Hardegree, Sheley et al., 2016). Despite the simplicity of wet‐thermal accumulation models, a relatively large amount of data and processing is required to develop the models and estimate seed germination timing in the field.

To overcome the logistical challenges associated with predicting seed germination timing, we created a programmed workbook called “Auto‐Germ” that allows users to efficiently process seed germination data and predict seed germination timing in the field. Our workbook utilizes Visual Basic for Applications (VBA) in Microsoft Excel (Microsoft Corporation, Redmond, Washington, USA) to create wet‐thermal accumulation models as well as calculate various other germination indices from laboratory constant temperature trials. Auto‐Germ also provides users with an interface to apply the wet‐thermal accumulation models to estimate germination timing in the field from historic soil moisture and temperature data sets.

Auto‐Germ's predictive germination modeling capabilities have the potential to educate practitioners in knowing how their planting dates may influence germination timing and subsequently the growing conditions that impact seedling establishment. The *Artemisia* spp. (sagebrush)‐steppe ecosystem in the Great Basin region of the western United States is an example of an imperiled ecosystem that would benefit from improved restoration practices (Hardegree, Jones et al., 2016; Suring, Rowland, & Wisdom, 2005). In this region, seeding is used to reclaim degraded sites that have been impacted by wildfires, invasive species, and various human disturbances (Davies, Bates, Madsen, & Nafus, 2014; Knick et al., 2011; Noss, 1995). In the *Artemisia*‐steppe, seeding typically occurs in autumn, with the expectation that seeds will remain dormant in the soil and then germinate in the spring (Crawford et al., 2004; Madsen, Davies, Boyd, Kerby, & Svejcar, 2016; Richards, Chambers, & Ross, 1998). However, planting too early in the year can result in seeds germinating prior to winter and then experiencing high mortality over the winter period (James & Svejcar, 2010). Winter mortality may occur as a result of freezing conditions (Boyd & Lemos, 2013; James, Svejcar, & Rinella, 2011). Roundy and Madsen (2016) determined that across 14 *Artemisia*‐steppe sites there was an average of 58 freeze–thaw periods for the upper 1–3 cm of soil between October and March. Seedbed freezing conditions have been shown to alter the physiological responses of *Artemisia tridentata* Nutt. (Asteraceae) (big sagebrush) in the Great Basin (Loik & Redar, 2003), and has the potential to further inhibit plant survival of perennial grasses such as *Pseudoroegneria spicata* [Pursh] A. Love (bluebunch wheatgrass) (Boyd & Lemos, 2013). Mortality may also occur to seedlings over the winter period as a result of drought, pathogens, and expenditure of seed carbohydrate resources (James et al., 2011; Madsen et al., 2016). Subsequently, in this region understanding the seeding date required to prevent premature germination and subsequent winter mortality is paramount to improve the effectiveness of restoration projects.

Our objectives were to provide instructions on how to use Auto‐Germ and demonstrate the utility of the program through a case study that (a) calculated various germination indices under different constant temperatures on 10 different species commonly used for restoration projects in the Great Basin and (b) for these same species model seed germination timing across 6 years and 10 *Artemisia*‐steppe sites to estimate the planting date required for 50% or more of the simulated population of seeds to germinate in spring (March 1st or later) when conditions are predicted to be more conducive for plant establishment.

## 2 METHODS AND MATERIALS

### 2.1 Instructions for operating auto‐germ

Auto‐Germ can be downloaded at [https://autogerm.byu.edu/]. There are four main steps for processing data in Auto‐Germ, which include: (a) entering laboratory data, (b) wet‐thermal model creation, (c) entering field data, and (d) model application. Each step is initiated by clicking a button in Auto‐Germ on the Home worksheet (note macros and content must be enabled to use Auto‐Germ). Auto‐Germ provides instructions on the Home worksheet for each step (Supporting information Figure S1).

#### 2.1.1 Step 1—Germination count data input

The first step is to input germination count data from constant temperature laboratory trials into the Data Entry worksheet (Supporting information Figure S2), which is accessed by clicking the Data Entry button. To input new data, click the Start Over button on the Data Entry worksheet. In Auto‐Germ, the data organization must match the sheet setup, where column A is temperature in Celsius, column B is replicate (or block), column C is plot ID, column D is treatment, column E is the number of seeds planted per sample, and everything from column F to the right is measurement dates and their respective germination counts. The planting date is entered into cell B8. The workbook processes up to 100 germination date entries and 1,000 samples. Under each measurement date, enter the number of seeds that germinated between the last count time and the current one. Do not enter cumulative germination count data on this sheet. Entries in the columns labeled as rep/block and plot ID are optional. If the user does not want to produce wet‐thermal accumulation models, germination metrics will be calculated through Auto‐Germ without temperature data. Auto‐Germ will not operate if empty cells are included under the columns labeled as temperature, treatment, seeds planted, planting date, and the germination measurement columns. The treatment column can be used to signify a number of different variables. For example, if seed treatments are being analyzed the type of seed treatment would be placed in this column. If species were being compared the treatment column would contain the name of the species.

#### 2.1.2 Step 2—Wet‐thermal model creation

Once the data is entered, return to the Home worksheet and click the Make a Model button, and enter in the pop‐upwindow the lower and upper germination percentage and interval size to model. The workbook can model any range of germination percentages from 1% to 99%. The four new worksheets created are called Germination Metrics, Data Averages, Standard Error, and Polynomial Equations. Once the calculations are completed, a pop‐up window notifies that the data are ready to be viewed. Click the View Data button under the Workbook Options heading to view the worksheets in a new workbook that can be saved, or click the worksheet tabs on the bottom of the screen. The Germination Metrics sheet displays the whole data set sorted by treatment, temperature, and calculated germination metrics. The calculated metrics for each sample include the number of seeds that germinated, final germination percentage, mean germination time, coefficient of variation of the germination time, mean germination rate, uncertainty of germination, synchrony of germination, and time to reach each percent germination (Ranal, Santana, Ferreira, & Mendes‐Rodrigues, 2009).

Mean germination time is calculated as:

(1) $$\overset{¯}{t}=\frac{\sum_{i=1}^{k}n_{i}t_{i}}{\sum_{i=1}^{k}n_{i}}$$

where $\overset{¯}{t}$ = mean germination time;*t*_*i*_ = time from the start of the experiment to the *i*th observation; *n*_*i*_ = number of seeds germinated in the *i*th time; *k* = last time of germination.

The coefficient of variation is calculated as follows:

(2) $${\text{CV}}_{t}=\frac{s_{t}}{\overset{¯}{t}}\times 100$$

where *CV*_*t*_ = coefficient of variation of the germination time; *s*_*t*_ = standard deviation of the germination time; $\overset{¯}{t}$ = mean germination time.

The mean germination rate is calculated by taking the inverse of the mean germination time. The uncertainty of germination is calculated as:

(3) $$U=-\sum_{i=1}^{k}f_{i}\times \log_{2}f_{i}$$

where *U *= uncertainty of the germination process

$$f_{i}=\frac{n_{i}}{\sum_{i=1}^{k}n_{i}}$$

*n*_*i*_ **= number of seeds germinated on the *i*th time;*k *= last time of observation.

The synchrony of germination was calculated as follows:

(4) $$Z=\frac{\sum_{i=1}^{k}C_{n_{i},2}}{C_{\sum n_{i},2}}$$

where *Z* = synchrony of germination

$$C_{n_{i},2}=\frac{n_{i}\left(n_{i}-1\right)}{2}$$

$C_{n_{i},2}$ = combination of the seeds germinated in the *i*th time, two by two; *n*_*i*_ = number of seeds germinated on the *i*th time.

The time to reach each percent germination was calculated as follows:

(5) $$T_{N}=\left[\left(\frac{t_{a}\;-t_{b}}{n_{a}-n_{b}}\right)\;\left(N-n_{b}\right)\right]+t_{b}$$

where *T*_*N*_ = time (days) to subpopulation germinatio; *t*_*a*_ **= incubation day when subpopulation germination was reached; *t*_*b*_ = incubation day before subpopulation germination was reached; *n*_*a*_ = number of germinated seeds on day that subpopulation germination was reached; *n*_*b*_ **= number of germinated seeds on day before subpopulation germination was reached; *N * =  number of germinated seeds equal to the percentage of the total subpopulation of interest.

The Data Averages worksheet displays the same metrics for the average of each treatment and temperature combination. The Standard Error worksheet displays the standard error for each calculation on the Data Averages worksheet. The Polynomial Equations worksheet contains second order polynomial equations with their associated coefficient values (A, B and C), the *R* ^2^ value for each germination percentage of each treatment, and the corresponding graphs depicting germination rate as a function of temperature (Supporting information Figure S3). To create new polynomial equations the newly created sheets need to be exported or deleted.

#### 2.1.3 Step 3—Field data input

To estimate seed germination timing in the field from the polynomial equations, the user needs to create worksheets containing field soil temperature and water potential data. Click the See Sample Data button on the Home worksheet to see how field data worksheets should be formatted. Create separate worksheets for separate sites and planting years. The format of the data must match the example data in the worksheet, where column A is the measurement date and time, column B is temperature, and column C is water potential. The user must input their own field data worksheets to apply the model. The field data worksheets must be located in‐between the Home and Data Entry worksheets. If there are any other worksheets besides field data in this location, the program will not operate correctly.

#### 2.1.4 Step 4—Field germination predictions

At this point, two options are available for the user to choose from. The first option is to predict the time to reach the previously specified germination percentages based on a planting date. The second option is to predict the dates a certain germination percentage is reached based on a range of planting dates. Before clicking either button, make sure that steps 1–3 are complete and that the Polynomial Equations worksheet is located in the workbook somewhere after the Data Entry worksheet. If Polynomial Equations are missing or has a changed name, Auto‐Germ will not operate.

To predict the times to reach the previously specified germination percentages, click the Choose Planting Date button on the Home worksheet. Enter the planting date to model for in the pop‐up window. The minimum water potential threshold can be changed from the default value of −1.5 MPa, based on the species being evaluated. The new worksheet created is named Planting Date (Supporting information Figure S4). The tables on the left of Planting Date show the predicted dates when the corresponding germination percentages will occur for each treatment according to each individual field data sheet. The graphs of the tables are located on the right.

To predict the dates a certain germination percentage is reached, click the Choose Germination Percentage button on the Home worksheet. Enter the percent germination and the range of planting dates to model in the pop‐up window. The minimum water potential threshold can also be changed from the default value of −1.5 MPa. The new sheet is named % Germination (Supporting information Figure S5). The tables on the left of % Germination show the predicted time to reach the specified percent germination, given the specified range of planting dates. Each table corresponds to a field data sheet. The graphs of the tables are located on the right.

#### 2.1.5 Workbook Options

Workbook Options is the last heading on the Home sheet. The View Data button will create a new workbook that contains all of the data generated from steps 2 and 4, but will not remove any new worksheets. The new workbook containing generated data may be saved. The Export Data button will export the data that was generated in steps 2 and 4 to another workbook that can be saved, and data will be removed from Auto‐Germ. The Start Over button will completely reset Auto‐Germ and delete all the data generated, but will not affect worksheets located before Data Entry.

### 2.2 Case study

#### 2.2.1 Laboratory methods

We developed wet thermal‐time models for 10 seedlots of species commonly used in restoration projects in the Great Basin. We included eight perennial grasses; *P. spicata, Leymus cinereus* (Scribn. & Merr.) Á. Löve (Great Basin wildrye)*, Festuca idahoensis* Elmer ssp. *Idahoensis* (Idaho fescue)*, Poa secunda* J. Presl (Sandberg bluegrass), *Elymus wawawaiensis* J. Carlson & Barkworth (Snake River wheatgrass), *Elymus lanceolatus* (Scribn. & J.G. Sm.) Gould (thickspike wheatgrass), and *Elymus elymoides* (Raf.) Swezey (bottlebrush squirreltail), two forb species; *Linum lewisii* Pursh (Lewis flax) and *Achillea millefolium* L. var. *occidentalis* DC. (western yarrow)*,* and one shrub species; *Artemisia tridentata* Nutt. ssp. *wyomingensis* Beetle & Young (Wyoming big sagebrush). Seed was purchased from certified lots at Granite Seed (Lehi, UT, USA). A range of constant temperatures was used to germinate the seeds (5, 10, 15, 20, and 25°C). The study was setup using a randomized block split‐plot design, with temperature comprising the split plot. Seven repetitions were used for each species, at every temperature. In each repetition, 25 seeds were placed in a 9 cm diameter petri dish that contained a single layer of blotter paper. Five ml of water was initially added to each petri and additional water was added as petri dishes dried throughout the study. Petri dishes were closed in plastic bags by block to prevent the loss of water. Germinated seeds were counted every 1–3 days, for 60 days. Seeds that had germinated were counted, recorded, and removed from the petri dishes. Germination count data was then processed in Auto‐Germ.

Auto‐Germ was used to calculate final germination percentage, *T*_50_, synchrony, and mean germination time. We then used mixed model analysis in JMP^®^ (Version 13, SAS Institute Inc., Cary, NC, USA) to first determine the significance (*p *≤ 0.05) of these four indices with respect to species, incubation temperature, and their interactions (unless determined to not be significant). In the model, blocks were considered random, while incubation temperature and species were both considered fixed. We tested for differences in responses to species at the incubation temperatures of 5, 10, 15, 20, and 25°C using a Tukey pairwise comparison test (*p *≤ 0.05). Final germination was squared and the log of *T*_50_, synchrony, and mean germination time was taken to normalize the data.

#### 2.2.2 Field germination predictions

Wet‐thermal accumulation models for each species was applied to historical soil temperature and water potential data from the Sagebrush Step Treatment and Evaluation Project (SageSTEP) (Cline, Roundy, & Christensen, 2018a, 2018b) to determine how planting date influenced germination timing. We selected from the SageSTEP network ten different sites to model seed germination timing that were within *Artemisia*‐steppe and *Pinus* spp.‐ *Juniperus* spp.(pinyon‐juniper) woodland communities that had been treated with prescribed burns (Moses Coulee, WA, Saddle Mountain, WA, Bridge Creek, OR, Hart Mountain, OR, Marking Corral, NV, Owyhee, NV, Blue Mountain, CA, Greenville Bench, UT, Onaqui, UT, and Stansbury, UT) (McIver & Brunson, 2014). At each of these sites, hourly measurements were made at approximately 1–3 cm below the soil surface to estimate soil temperature using thermocouples and soil water potential using gypsum blocks (Delmhorst Inc., Towaco, NJ, USA).

At each of the field sites, we evaluated seed germination timing for each of the 10 seedlots using the second option in Step 4 on the Home worksheet, which predicts the dates a certain germination percentage is reached based on a range of planting dates. Simulations were ran on 6 different years with daily planting dates between September 1st and March 1st. For each simulated planting date, we analyzed for the date a simulated population of seed would reach 50% germination. A base water potential threshold of −1.5 MPa was used in the simulations, based off of previous studies (Rawlins, Roundy, Davis et al., 2012; Rawlins, Roundy, Egget et al., 2012).

We used the planting date required for 50% or more of the simulated population of seeds to germination in spring (i.e., 1 March or later) as the metric to compare between species. This metric was chosen because it is estimated to be the planting date required for land managers to circumvent the limiting biotic and abiotic factors causing mortality to seedlings during the winter. We used mixed model analysis to first determine the significance (*p *≤ 0.05) of species, site, and year for germination date (all fixed variables). We then tested for differences in responses to species, site, and year using a Tukey pairwise comparison test (*p* ≤ 0.05).

## 3 RESULTS

### 3.1 Germination indices

**Figure 1:**
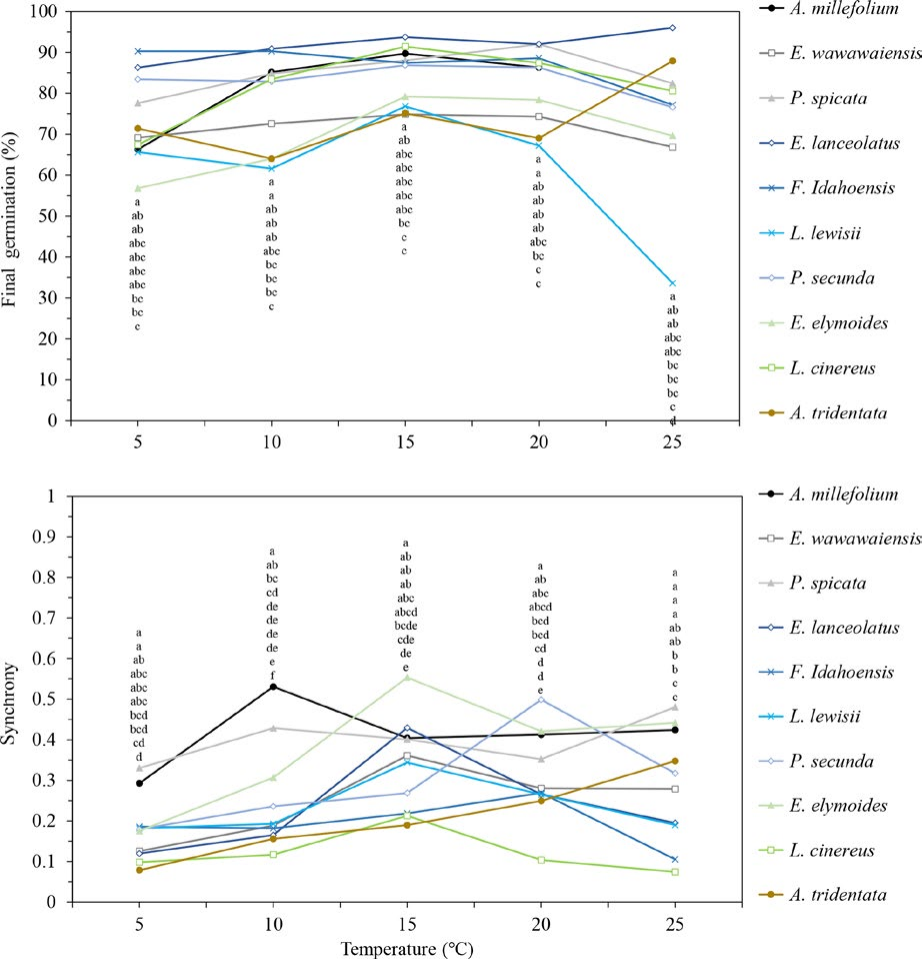
Final germination percentage and synchrony at temperatures ranging from 5–25°C for 10 different species commonly seeded in the Great Basin, USA. Values with the same incubation temperature with different letters are significantly different (*p *≤* *0.05) at that temperature. Letters correspond with the order of the data points in the figure

Incubation temperature, species, and the interaction between these two factors affected final germination percentage (*F* = 10.5, *p *<* *0.001; *F* = 23.6, *p *<* *0.001; *F* = 2.9, *p *<* *0.001), synchrony (*F* = 49.0, *p *<* *0.001; *F* = 52.6, *p *<* *0.001; *F* = 5.9, *p *<* *0.001), *T*_50_ (*F* = 1240.9, *p *<* *0.001; *F* = 143.4, *p *<* *0.001; *F* = 25.6, *p *<* *0.001), and mean germination time (*F* = 726.8, *p *<* *0.001; *F* = 116.1, *p *<* *0.001; *F* = 18.8, *p *<* *0.001), respectively. As would be expected for cool‐season species in the Great Basin, germination was highest in general around 15°C and typically declined under the lowest (5°C) and highest (25°C) temperatures. The degree that germination percentage changed by temperature was variable for each species, with some species showing a limited change in germination with temperature (*E. lanceolatus, P. spicata*,* F. idahoensis*, and *P. secunda*), while other species were more variable (*A. millefolium, E. wawawaiensis*,* L. lewisii*,* E. elymoides*,* L. cinereus*, and *A. tridentata*; Figure 1). Subsequently, it was at the highest and lowest temperatures tested where there was the greatest range in germination between species. For example, at 25°C*, E. lanceolatus* had the highest final germination percentage (96%) and *L. lewisii* had the lowest (34%). At 5°C*, F. idahoensis* had the highest final germination percentage (90%) *while E. elymoides* had the lowest (57%; Figure 1).

Synchrony values fluctuated greatly between temperatures for all species (Figure 1). There were five species that had synchrony values above 0.40 (*E. lanceolatus, P. spicata*,* A. millefolium*,* E. elymoides*, and *P. secunda*). Both *L. cinereus* and *A. tridentata* consistently had the lowest synchrony values (0.08–0.18; Figure 1).

**Figure 2:**
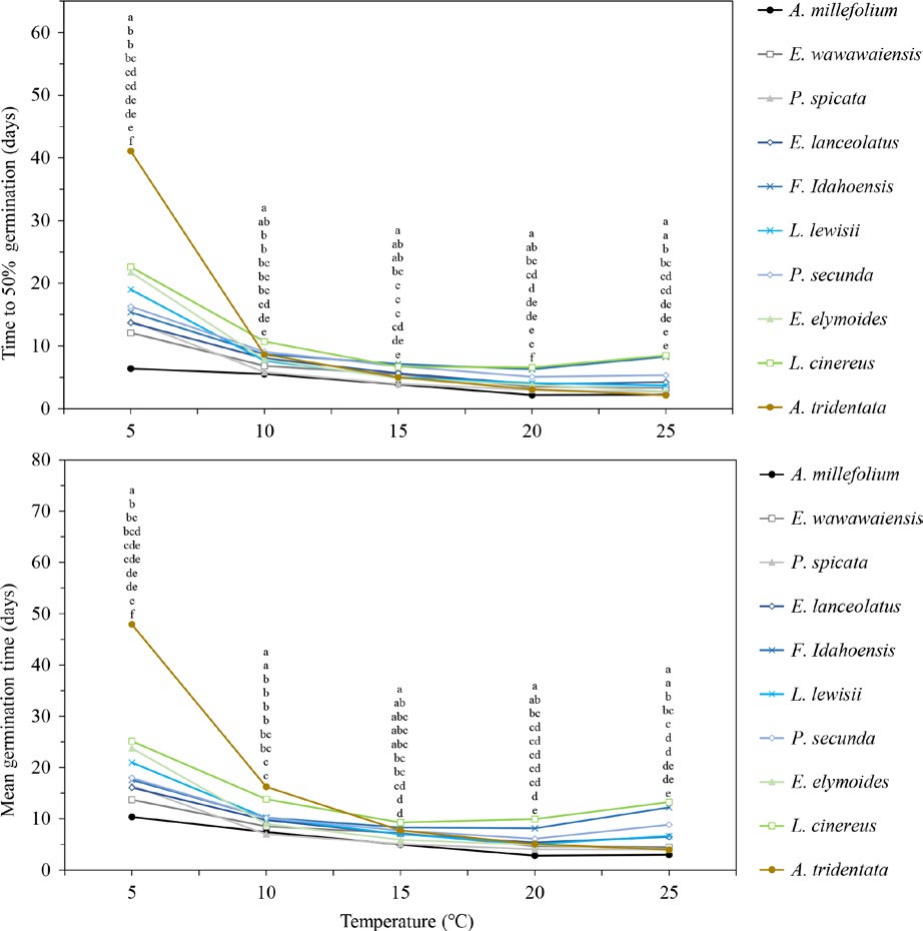
Time to 50% germination and mean germination time at temperatures ranging from 5–25°C. Values with the same incubation temperature with different letters are significantly different (*p *≤* *0.05) at that temperature. The letters correspond with the data points from top to bottom. Letters correspond with the order of the data points in the figure

Both T_50_ and mean germination time followed similar patterns, where all species had the highest values at 5°C, and then decreased until 20 and 25°C when many species had slight increases in germination time (Figure 2). The greatest difference between consecutive temperatures for both T_50_ and mean germination time occurred with *A. tridentata* between 5 and 10°C (32 and 31 days). Out of all the species, *A. tridentata* had the highest T_50_ and mean germination time at 5°C (41 and 48 days, respectively), but then these values quickly decreased as temperature increased; by 25°C, this species produced one of the fastest germinating times (2 and 4 days, respectively). *L. cinereus* had the second highest T_50_ and mean germination times at 5°C (22 and 25 days), but relative to the other species it maintained high values as temperature increased*. A. millefolium* was typically the fastest germinating species as shown by *T*_50_ and mean germination time values. However, at 10°C mean germination time was lower for *P. spicata* by 7 days and at 25°C, T_50_ was lower for *A. tridentata* by 2 days (Figure 2).

### 3.2 Field predictions

**Figure 3:**
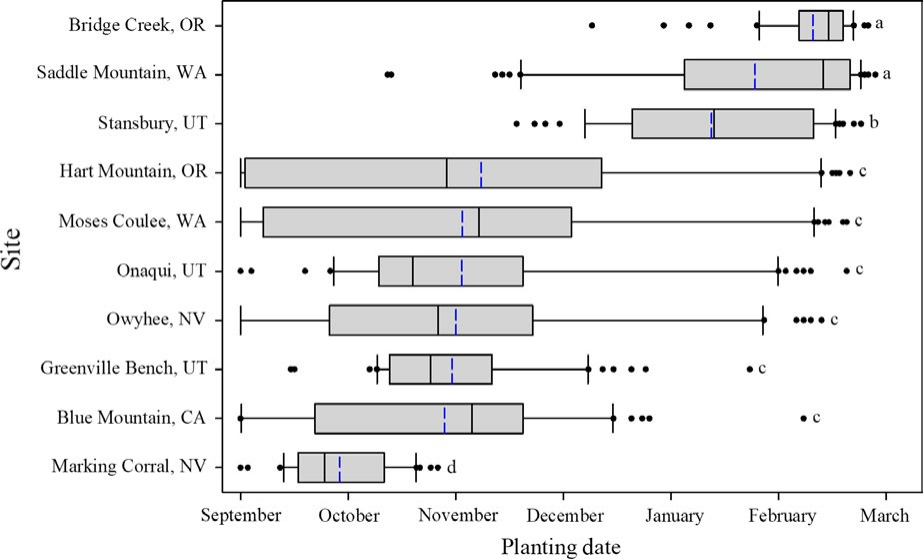
Planting date by site required for 50% or more of the simulated population to germinate in March or later. Box limits represent the first and third quartiles, the black line within the box indicates the median, the blue line indicates the mean, the whiskers’ limits represent the 10th and 90th percentiles, and the individual dots represent outliers. Plots with different corresponding letters are statistically different (*p *≤* *0.05)

**Figure 4:**
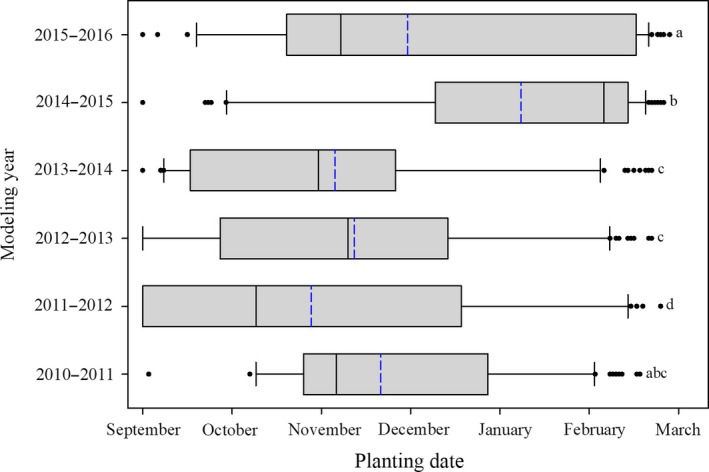
Planting date by modeling year required for 50% or more of the simulated population to germinate in March or later. Box limits represent the first and third quartiles, the black line within the box indicates the median, the blue line indicates the mean, the whiskers’ limits represent the 10th and 90th percentiles, and the individual dots represent outliers. Plots with different corresponding letters are statistically different (*p *≤* *0.05)

Wet‐thermal accumulation models appeared to have sufficient accuracy to predict germination time (adjusted *R* ^2^ = 0.71–0.98). Species (*F* = 23.2, *p *<* *0.001), site (*F* = 146.4, *p < *0.001), and year (*F* = 79.3, *p *<* *0.001) affected the planting date required to have 50% or more of the population germinate after 1 March. The site that produced the earliest average planting date across all species was Marking Corral (28 October), while the site that produced the latest average planting date across all species was Bridge Creek (7 February; Figure 3). Seven of the sites had average planting dates in mid‐fall to early winter (September–November), while the other three sites had average planting dates much later in the season (January–February; Figure 3). All years had similar ranges, with 2011–2012 having the earliest average planting date (27 October), and 2014–2015 having the latest (6 January; Figure 4).

**Figure 5:**
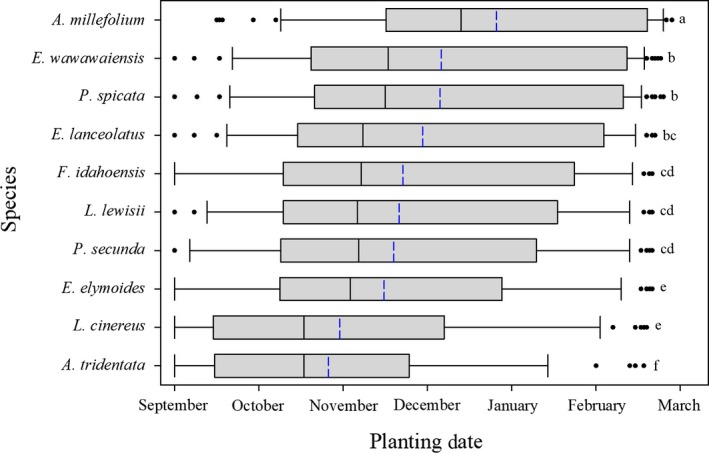
Planting date by species required for 50% or more of the simulated population to germinate in March or later. Box limits represent the first and third quartiles, the black line within the box indicates the median, the blue line indicates the mean, the whiskers’ limits represent the 10th and 90th percentiles, and the individual dots represent outliers. Plots with different corresponding letters are statistically different (*p *≤* *0.05)

Analysis by individual species showed each species had average planting dates as early as September, and as late as February to have 50% or more of the population germinate after 1 March (Figure 5). While there was extreme variability across all species in the date required for the majority of the seeds to germinate by spring or later, certain species consistently required later planting dates than others*. A. millefolium* had the latest average planting date (24 December), with the interquartile range of the data falling between 15 November and 16 February. The only other two species that had average planting dates in December were *E. wawawaiensis* (5 December) *and P. spicata* (4 December). These species, while having later average planting dates than all other species besides *A. millefolium*, had some of the largest interquartile ranges (19 October–9 February and 20 October–7 February respectively*). E. lanceolatus* (28 November*), F. idahoensis* (21 November), *L. lewisii* (19 November*), P. secunda* (18 November), and *E. elymoides* (14 November) all had average planting dates in November*. L. cinereus* (29 October) *and A. tridentata* (25 October) had the earliest average planting dates, with interquartile ranges that began in mid‐September (14 September, 15 September), and ended as early as late November—early December (23 November, 6 December; Figure 5).

## 4 DISCUSSION

Our case study demonstrates that Auto‐Germ has the potential to enable researchers to efficiently process laboratory germination data and field soil moisture and temperature data to predict various germination indices, including field germination timing. Based on these results, we anticipate that Auto‐Germ will be applicable to non‐dormant seeds of most species. Both land managers and researchers could benefit from this program by providing them with a better understanding of how seeds may respond to their planting sites’ unique soil temperature and moisture regimes.

It should be noted that predictions developed from Auto‐Germ should be used as rough assessments to help guide further research and management. Wet‐thermal models used in Auto‐Germ can overestimate germination rates (more so than other hydrothermal models) but these errors are expected to be minimal (Hardegree et al., 2017; Rawlins, Roundy, Egget et al., 2012). In previous studies that have validated wet‐thermal accumulation models, non‐linear regression equations were used from TableCurve 2D (Systat Software Inc., San Jose, CA, USA) curve‐fitting program (Rawlins, Roundy, Davis et al., 2012; Rawlins, Roundy, Egget et al., 2012; Roundy et al., 2007). In these studies, the *R* ^2^ values of the models ranged from 0.70 to 0.98. For our case study, a more simplified second order polynomial was used to allow processing in Microsoft Excel. This study indicated that second order polynomials provided a similar level of accuracy to predict germination timing as other models (*R* ^2^ = 0.71–0.98).

The germination indices calculated showed that individual species react uniquely to differences in soil temperature (Figures 1 and 2). For example, *A. tridentata* at 5°C had an extremely high T_50_ and mean germination time in relation to the other species tested (almost 2× more than *L. cinereus*, the species with the next highest values; Figure 2). However, as the temperature increased, *T*_50_ and mean germination time decreased to levels similar to the other species. Given this information, it is impractical for land managers to plant different species at the same date and expect similar results in germination timing.

Our case study also showed how these unique germination characteristics affected when species would germinate in the field under historic soil moisture and temperature settings (Figures 3, 4, 5). Auto‐Germ was used to calculate when 11 different species would need to be planted to have the majority of germination occur after 1 March, across 6 years, and 10 *Artemisia*‐steppe sites in the Great Basin of North America. Looking at all species collectively by site showed that the required planting date for germination to occur after 1 March was highly variable, with planting dates ranging from September to February, due to differences in the sites soil temperature and moisture (Figure 3). The year of planting was also highly variable when looking at all species collectively by planting year, with required planting date for germination to occur after 1 March ranging from November ‐January (Figure 4). Additionally, on a species basis, there was high variability between some species with respect to the planting date that would allow germination to occur after 1 March. In general, we found that species that exhibited lower *T*_50_ and mean germination time values (particularly under colder temperatures), such as *A. millefolium*,* E. wawawaiensis*, and *P. spicata* (Figure 2)*,* on an average all required planting dates by December for the majority of the simulated population to germinate after 1 March. Conversely, species with higher T_50_ and mean germination time values, such *as L. cinereus* and *A. tridentata,* could be planted much earlier in the season (October), and typically not have the majority of the seeds germinate over the winter.

Two key points can be taken from this portion of the study, firstly that restoration plans developed for a species at one site or year do not translate to sites and years with different soil temperature and moisture regimes. The optimal planting date (the date required for the majority of germination to occur after 1 March) for a species varies greatly between sites where the climates are different. The same principle can be applied to variability seen on a year to year basis. The annual environmental changes at individual sites create vastly different results for planting dates. The second key point is that at any given site, understanding the germination characteristics of individual species may increase the success rates of restoration projects. For example, planting *A. tridentata* in mid‐October may be late enough in the season to circumvent winter germination at multiple sites; however, for a species such as *P. spicata*, which germinates more quickly, a planting date in mid‐December might be more suitable.

These differences between species germination timing (Figure 2 and 5) may be beneficial when applied to bet‐hedging strategies surrounding seed mixes. Rinella and James 2017 predicted that seed mixes of *both P. spicata and P. secunda* would lead to better establishment than individually seeded species. As shown from the germination indices calculated in this study, the species used reacted in unique ways to different temperatures, both in the timing and spread of germination. This demonstrates how individual species may be better suited for different sites and their relative suitability may change depending on the planting year. Using multiple species with different germination characteristics could decrease the risk of seeding failure by spreading the period that seeds germinate under and thus increase the probability of having some of the species in the mix germinate during a period that is favorable for plant establishment.

Our findings provide evidence that winter mortality may play a role in the lack of spring emergence seen in restoration efforts due to species germinating prior to or during the winter period and being subjected to freezing conditions. For all species *except A. tridentata* and *L. cinereus*, 50% or more of the required planting dates for spring germination occurred by November or later. This means that land managers who seed areas in mid to late fall would run the risk of having germination occur outside of more favorable spring conditions. Premature germination could potentially be mitigated by planting later in the season, however this study shows that seeding would need to take place in early to late winter. Winter seeding can be logistically challenging due to freezing and/or saturated soil conditions impacting the delivery of seed from mechanical equipment. One potential solution may be to treat the seeds and induce seed dormancy over the winter period. Richardson (2018) demonstrated that seed dormancy can be induced through the addition of the plant hormone abscisic acid (ABA), which is applied to the seed through a seed coating. It may be possible to have seeds that are not suitable for planting in early fall treated with an ABA seed coating so that the seeds germinate in spring when conditions may be more favorable for plant establishment and growth.

## 5 CONCLUSION

Our research indicates that Auto‐Germ provides researchers with a tool to efficiently model germination timing to understand the germination patterns of species across large temporal and spatial spectrums. As shown through our case study in the Great Basin, Auto‐Germ was able to generate germination indices and predict seed germination timing in the field, over six different years, for 10 different species commonly used for restoration projects. The results of this research provide new insights into when these species should be planted and can help guide scientists and land managers in developing new restoration technologies and practices.

## CONFLICT OF INTEREST

None declared.

## AUTHOR'S CONTRIBUTIONS

MM, DW, KS, and NB conceived the ideas and helped program Auto‐Germ. WR and RC collected and analyzed the data. BR collected field soil moisture and temperature data. WR, MM, BR, and ZA helped write and organize the manuscript. All authors contributed to the editing of the drafts and gave final approval for publication.

## DATA ACCESSIBILITY

Data are accessible via the Dryad Digital Repository, https://doi.org/10.5061/dryad.r6d4190.
