# Supplementary material for: Use of auto‐germ to model germination timing in the sagebrush‐steppe
Source: Ecol Evol. 2018 Nov 14;8(23):11533–42. doi: 10.1002/ece3.4591 (PMC6303710; doi:10.1002/ece3.4591)
Supplement: Supplementary file 1 [file ECE3-8-11533-s001.docx]

## APPENDIX


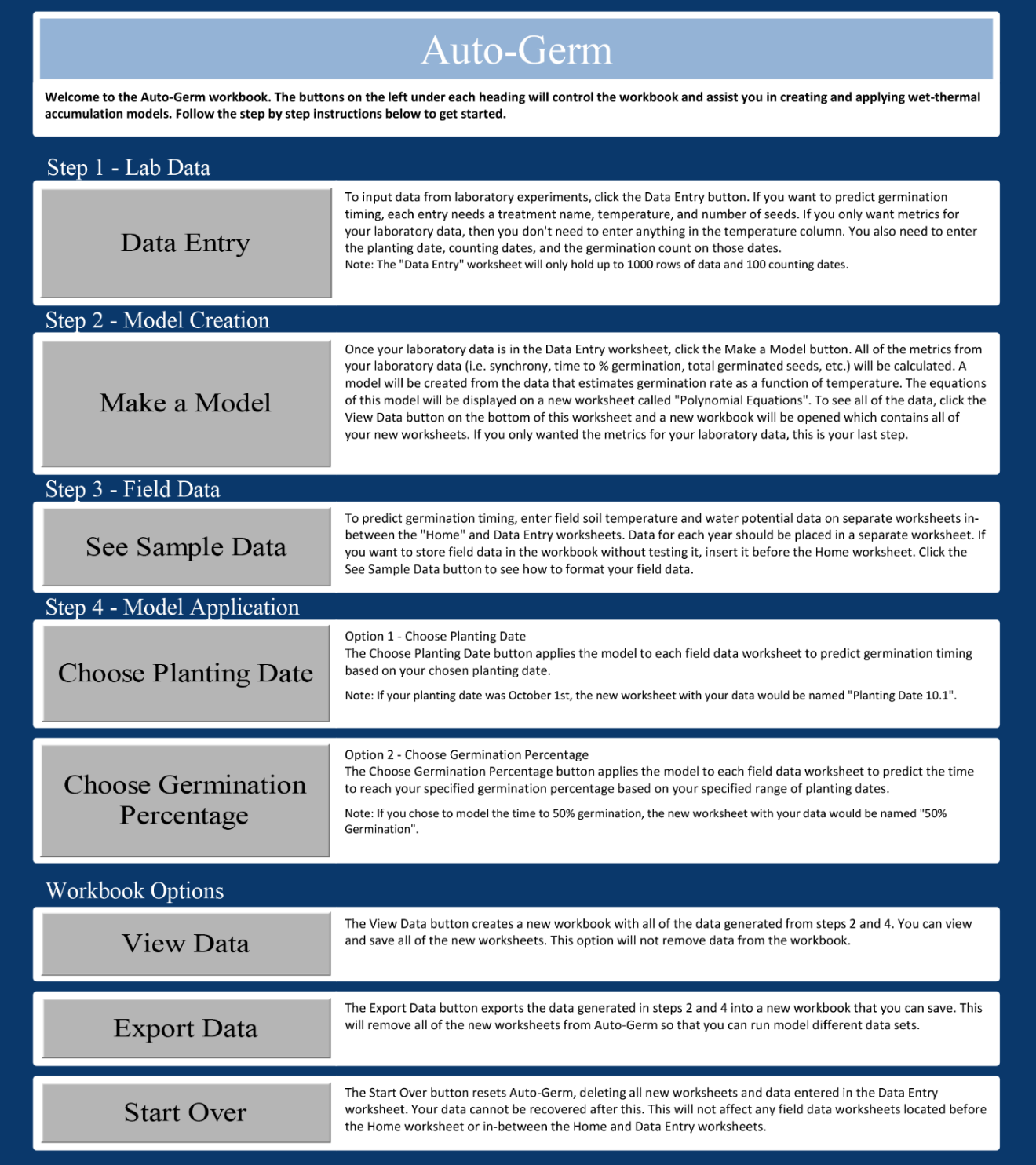


Figure S1. The first or “Home” worksheet in Auto-Germ. This worksheet has step by step instructions on how to use each feature of the workbook.


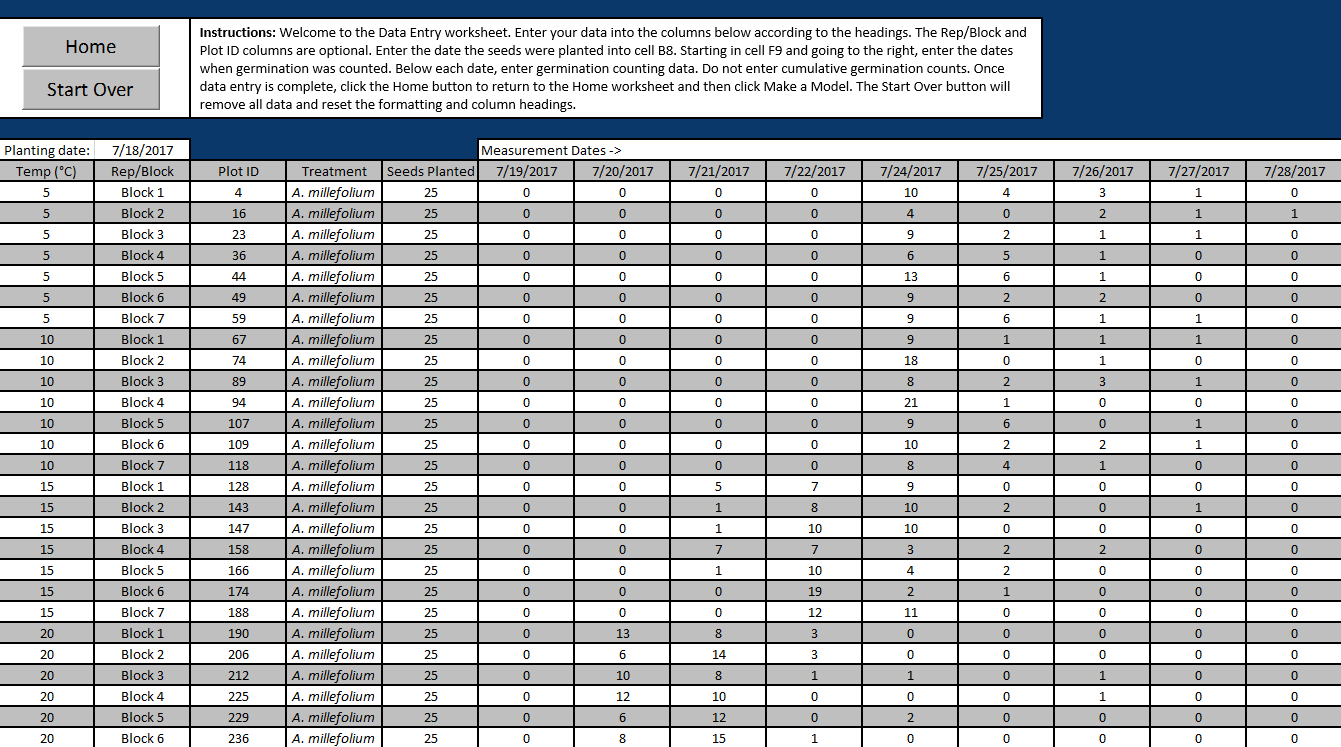


Figure S2. A completed example of the “Data Entry” worksheet in Auto-Germ. This is where germination count data is input from constant-temperature laboratory trials.


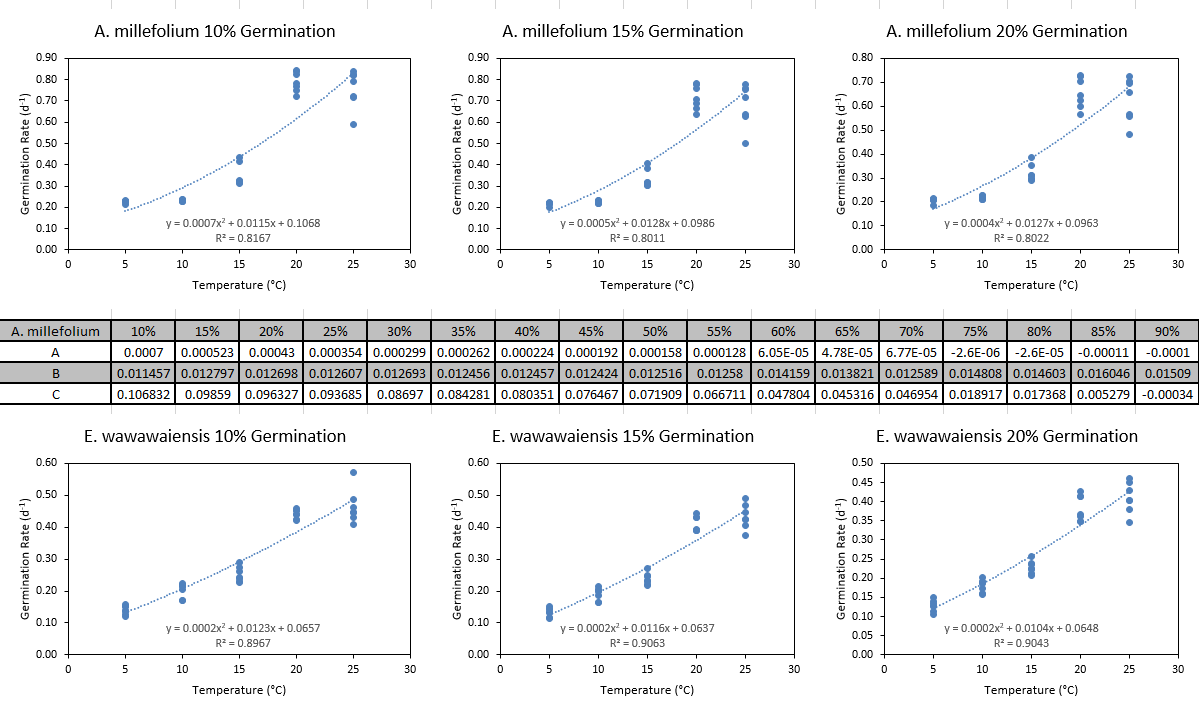


Figure S3. A completed example of the “Polynomial Equations” worksheet. This sheet contains the coefficient values and graphs of the second degree polynomial equations for each treatment and percent germination combination.

Figure S4. An example of the figures found in the “Planting Date” worksheet. Shows the predicted germination times of six species common to the Great Basin of North America, based on soil temperature and water potential data from Hart Mountain, OR for the year 2010. The simulated planting date was on October 15^th^.

Figure S5. An example of the figures found in the “% Germination” worksheet. Shows the date at which the simulated population will reach 50% germination for six species common to the Great Basin of North America, for every planting date between 10/15/2010 and 01/15/2011 at Hart Mountain, OR.
